# Anatomical and Physiological Observations Made upon Decapitated Subjects

**Published:** 1869-07-15

**Authors:** Ch. Robin

**Affiliations:** From Robin’s Journal of Anatomy and Physiology


### CHICAGO MEDICAL JOURNAL.

Vol. XXVI.—JULY 15, 1869.—No. 14.

### TRANSLATIONS.

### *Anatomical and Physiological Observations made upon Decapitated Subjects.*

BY M. CH. ROBIN.

*From Robin's Journal of Anatomy and Physiology.*

[Continued from Last Number.]

III.—State of the Arteries and Veins of the Trunk.

In the three decapitated subjects who were carefully studied with a view to determine the state of the vessels, we found the vertebral artery and vein in the trunk incompletely retracted upon themselves, containing air only, and showing the orifice occasioned by their transverse section, with margins a little depressed but open.

The two primitive carotids, incompletely retracted upon themselves, contained air, and air mingled with frothy blood was found throughout the whole extent of the sub clavian of each side, as well as in the origins of some of the arteries which originate therefrom.

The arch and a portion of the thoracic aorta, whilst very distinctly retracted upon themselves, contained frothy blood in which the volume of air exceeded, considerably, that of the blood with which it was mingled.

I have met with no author who has indicated the presence of air in the arteries of the head, which was referred to in the last paragraph. But it is interesting to compare with the facts which we have just mentioned, the following, which have been observed, at Brest, by Professor Marcellin Duval and by his assistants.

In two criminals subjected to experiment five or six minutes after decapitation, the extremities of the primitive carotids rose by regular pulsations, extended themselves, and protruded beyond the level of the plane of section of the neck, to retract again upon themselves.

At each impulse a small quantity of frothy scarlet blood escaped from the distended opening of the carotids. Blood was poured out, likewise, by the internal jugular veins. It was *equally frothy* but of a deeper color (M. Duval, *Expe riénces sur des supplicées.* Congrés Médical International de Paris, 1868, 8vo, p. 624).

It is very certain from the preceding facts, that air penetrates into the carotid arteries, in consequence of the tendency to the formation of a vacuum induced by the gradual retraction of the lung, although it could only enter therein during the short duration of each period of the cardiac diastole.

In the case of the first criminal whose vessels we dissected with the view to determine the nature of their contents, the left primitive carotid had been torn rather than cut. Its divided extremity was a little conical, ragged on the side of the trunk. There was a clot between the lips of the arterial section, and in the whole extent of the arm, the vessel and its branches, slightly retracted upon themselves, were filled with blood, by the side of the corresponding veins which were nearly empty. On the contrary, in the other limbs, and in the trunk, the arteries completely contracted upon themselves were bloodless.

In the walls of the trunk and in the limbs, the tissues are generally bloodless; but there remains, however, a little blood in the satellite veins of the arteries which themselves contain none. There is always more or less of it from one vein to another. Thus the testicular, diaphragmatic, mammary, internal and pericardiac veins contain, ordinarily, more than the satellite veins of the arteries of the limbs and of the abdominal parietes.

As to the internal jugular veins, the axillary, sub-clavian, brachio-cephalic and superior cava, they are found completely full of air. Here the air has naturally replaced the blood which flowed out from the internal jugular, by reason of the state of dilatation in which they are maintained by their adhesions to the aponeuroses and other organs to which they are attached, as has been clearly indicated by P. Bérard (*Archives Général de Médicine*, Paris, 1830, t. XXIII, p. 170.)

The auricular appendages, the right auricle and the ventricle were full of air, in two subjects, and the pulmonary artery contained black blood with some large bubbles of air. In the third, the auricle, the auricular appendage, and the ventricle of the right side contained very frothy blood, in which the air represented a mass much more considerable than the liquid.

We shall recur, in the following paragraph, to this point, which is only referred to here in order to mention that in two instances out of three, there were bubbles of air separating the little columns of blood in the cardiac veins, up to the point where their calibre diminished to a millimetre or less.

In all the subjects, there was gas in the trunk of the vena-cava, and in one of them there were seen big bubbles mingled with blood up to the level of the venal veins, and in the trunk of the hepatic veins.

The vena porta contained blood in all the subjects observed, as well as in the principal trunk, but always sensibly less than in the bodies of individuals dead from disease. This last fact is easily understood, by reason of this, that the blood of the mesenteric arteries, in place of being driven into the capillaries, thence into the corresponding veins by the arterial retraction consecutive to death escapes, in these decapitated subjects, from the direction of the aorta, and in the cavity of this conduit which empties itself through the several carotids and vertebrals.

The indication of the presence of air in the veins of the neck and of the heart of decapitated subjects, obliges us to notice carefully the absence of gas in the portal vein. In some animals, indeed, such as the plagiostome fishes, there occurs, in the blood of the vena-cava and hepatic veins, a spontaneous disengagement of gas in sufficient quantity to distend the vessels. This separation commences immediately after the death, by asphyxia, of the animal removed from the water (Voyez Ch. Robin, art. Hygrologie, *Dictionnaire d'Histoire Naturelle d'Orbiquy*, Paris, 1868, 2d edit., 8vo, t. VII, p. 489, et Suiv., et *des Tissues et des Secretions*, Paris, 1869, 8vo., p. 100.

The blood of the splenic vein contained leucocytes in quantity nearly double of that which was seen in drops of the same volume of blood from the splenic artery and from the pelvic veins.

It was easy for us to determine that water and acetic acid acted upon these leucocytes of the splenic veins, as upon those of blood, of pus, of mucus, etc. The action of these liquids was, on the contrary, quite different; that is, it was almost null upon the spherical nucleated epithelia obtained by compressing a little of the parenchyma of the spleen, so as to press and force out, in some measure the splenic pulp mingled with the blood in the large vessels of the organ.

The hepatic veins contained more blood, especially when compared with the hepatic branches of the portal vein.

IV—State of the Heart and Pulmonary Vessels.

In two decapitated subjects observed in my laboratory, the right auricular appendage and auricle were entirely filled with air. They were depressed since they had been cut, and their internal surfaces slightly moistened with blood. In these subjects there were bubbles of air separating little columns of blood, in the cardiac veins, as has been previously observed.

The right ventricle, laid open, collapsed, contained only a little frothy blood, formed of nearly equal volumes of air ond liquid.

The pulmonary artery and its branches, perceptibly retracted upon themselves, contained black blood, without air bubbles, in one of these subjects.

In the two otner subjects, the pulmonary artery was found in the same condition; but the blood, although dark colored, was mingled with a number of air bubbles, whose presence it was easy to recognize, particularly in the arterial trunk.

In one of the three subjects, the auricular appendage as well as the auricle and ventricle of the right side, very sensibly collapsed, contained only frothy blood, formed of almost equal volumes of gas and blood. This subject was one of those in whom bubbles of air were mingled with blood in the trunk of the pulmonary artery; the cardiac veins enclosed none of it.

After what has been said above of the conditions which favor the penetration of air into the superior cava, it must be understood that the respiratory movements cease after the section of the neck; the repletion of the right auricular appendage with air is inevitably favored by the gradual retraction of the lung, a retraction completed simultaneously by the entrance of the air into the veins and into the right heart above, by the ascent of the diaphragm and of the sub-diaphragmatic organs below. In every case, the left auricular appendage was entirely empty, although there was a little non-coagulated blood in the pulmonary veins.

The left ventricle formed a firm mass larger than that of the right, whose walls remained soft and compressible. In all, the left ventricle, strongly retracted upon itself, was empty, with the exception of a small quantity of blood, which was found, as it were, closed up in the cul-de-sac formed by the inter-ventricular space and the corresponding segment of the mitral valve. In one of them this blood, already coagulated, formed a small mass, having about the superficial area of a silver five-franc piece. Its thickness was a little less than that of these pieces, especially towards the bottom of the cul-de-sac; it gradually became thinner as it neared the free edge of the valve, at which level it terminated.

The presence of this small quantity of blood in the left ventricle of the three decapitated subjects, proves clearly that the ventricles do empty themselves in a manner absolutely complete after each systole. *

In the youngest of the subjects observed there was, moreover, on the left, between the three large columnæ caræ of *the mitral valve* an irregular clot of the size of an almond. It was soft, black, without trace of fibrine which had been deprived of red globules. It appeared to have been the product of the last outpouring from the left auricular ap-

---

* No author whom I have consulted has spoken of the contents of the cavities of the heart in decapitated subjects, nor yet of the influence of their repletion upon the contraction of the parieties of this organ, a fact which will be considered in the next paragraph.

pendage into the already immovable ventricle, the appendage having itself received blood which had just traversed the lungs, after the cessation of all inspiratory movement. Hifflesheim, by showing that in the circulation *the sanguineous circle is not interrupted at any point** has placed, in clear relief the importance of the question whether in each ventricular systole the cavities of the ventricles empty themselves completely, a question already resolved negatively by Senac, Bartholin, I. Müller, etc., whom many phosiologists regard but little, and of whom Bérard has given a historical sketch (*Cours de Physiologie*, Paris, 1851, t. III, p. 643).

This importance has become greater still by the experimental demonstration of this fact, that the capacity of each ventricle exceeds, by a fifth or a fourth that of the corresponding auricular appendage (Hifflesheim et Ch. Robin, année 1864, de ce Recuil, p. 416). M. Chauveau has shown us also that at the end of each ventricular systole, there is under the valvular arch of the left and right heart of the horse a conical cavity which still contains a determinate quantity of blood. (Gazette Médicale, 1856, et Béraud, *Elements de Physiologie*, Paris, 1857, 2d edit., t. II, p. 287.

### V.—Experiments upon the Contractions of the Heart.

The heart of the first of the decapitated subjects, referred to in this sketch, was observed nearly seven hours after death. That of the second only ten hours after his execution. No experiment was attempted upon either of them. The most careful observation failed to detect any spontaneous movement of these organs, not more than from the contact of the fingers or the point of the scalpel.

Before reporting the observations which I made upon the

---

* Hifflesheim, *Journal de Chimie et de Physique* de Laurent Gerhardt et Nickles, in 8vo, 1849, *Comptes rendus et mene de la Societe de Physiologie*, Paris, 1854, in 8vo, p. 273, *Journal de l'Anatomie et de la Physiologie*, Paris, 1864, in 8vo, t, 1, p. 437.

movements of the heart of the third subject, I will state that M. Marcellin Duval has established the facts,as follows: "The chest was rapidly opened about seven minutes after the death of one of the criminals. Previous to the incision of the pericardium, the pulsations of the heart could be distinguished through the two layers of this membrane. At the moment of incision about forty grammes of limpid and slightly yellowish serum escaped.

"The heart exposed without any change in its relations presented the following phenomena:

"After a brief but perceptible period of immobility, the auricle was elevated abruptly, and separated from the aorta, which is exposed, then rapidly recedes and resumes its original position. The auricular appendage was exposed (as though distended with fluid). During its erection the auricle was elongated, and the fringes and indentations of its margin expanded, like the fingers of the hand, to be again approximated when the organ collapsed.

"The double movement, perfectly rythmical, was reproduced forty-eight times during the first minute; but it was soon retarded, and in the course of the fifth minute it occurred but seven times. In the case of the first subject (1850), the right auricle presented, during an hour and a quarter, energetic and regular movements or pulsations, which, at first, recurred forty-three or forty-four times per minute, and continued in spite of the removal of the stomach, the liver, the intestines, the diaphragm, and even the lungs." (M. Duval, *loc. cit.*, 1867, p. 524, et journal *la France Médicale*, Août, 1867.)

The heart of the third subject upon which I experimented, was exposed at seven o'clock in the morning, the execution having taken place at forty or forty-five minutes past four o'clock.

Experiments were made upon this subject in the presence and with the assistance of M. Dupré, Surgeon to l'Hôpital de Bourg (Ain), and of two internes of that hospital, who by their skill as anatomists, rendered me great assistance, for which I can scarcely be sufficiently grateful.

I have already stated that the left ventricle of the heart of this subject was retracted and hard; that the left auricle and appendage, entirely empty, were retracted; that the right ventricle, although containing a little frothy blood, formed a mass smaller in volume than the left ventricle. The right auricle and appendage, partly collapsed, contained, likewise, frothy blood.

These organs were altogether motionless, without contraction, even when pricked, or when the surface was lightly scratched with the finger, even whilst they were yet warm.

I had already learned, by other physiological experiments, that the principle of the normal conditions which determine the contraction of hollow organs with muscular walls, consists, to a certain degree, of a maximum repletion of their cavities without excessive distension of their walls, whatever may be the nature of the body which fills them, provided that it does not attack them chemically or mechanically.

After examining the condition of the heart left in position for some minutes, I inflated it with air, by means of a tube introduced into one of the brachio-cephalic venous trunks, whilst the vena-cava inferior was obliterated, by compression below the diaphragm.

As soon as the right auricle and its appendage had become prominent, in consequence of their complete repletion, with a little less perfect repletion of the right ventricle, these organs entered spontaneously into perfect rythmical contractions, to the number of sixty to sixty-four per minute.

These contractions commenced at the apex of the auricle, at which point they were vermiform, and of which they determined the erection in a very marked manner.

The contraction of this organ was both peristaltic and veriform, since it was propagated to the base of the auricle. All the corresponding portion of the auricular appendage, even, and at the same time its opposite extremity (which is a little above the level of the coronary vein), whilst its portion situated at the level of the mouths of the venæ-cavæ, was seized with a contraction which was propagated rapidly over the whole organ, until they met the others, near the attachment of the auricular appendage to the ventricle. Immediately upon the cessation of this contraction, toward the base of the ventricle, the right ventricle was seen to contract from the apex towards the base.

These ventricular contractions were very weak; did not determine a sensible diminution of the volume of the organ, except when at their beginning the surface was scraped lightly with the back of a scalpel, and after a few systolic contractions, remained confined to a portion only of the right ventricular walls near its extremity.

Those of the auricular appendage, on the contrary, were energetic, as if accompanied by an effort to expel its contents, which did not occur under the experimental conditions assumed; for these compelled us to maintain the obliterations of the vessels emptying into the right side of the heart, so that the auricular appendage only collapsed during each systole proportionally to the compression which it experienced from the air. Hence, the elasticity of the air was the cause of the auricular diastole.

In spite of the precautions, at the end of eight minutes the auricular appendage and the ventricle were perceptibly emptied. The contractions of the appendage became convulsed, that is to say, interrupted, tremulous, very frequent, although continuing to accomplish themselves from the auricle towards the base of the ventricle, according to the order indicated above. The insufflation of an additional quantity of air having reduced the appendage to its original state of repletion, the contractions resumed their regularity. At the end of three or four minutes the right heart having been emptied of a portion of its gaseous contents, the contractions assumed, immediately, the interrupted character which has already been described, and which simulated to the eye, those which may be supposed to exist in certain palpitations of the heart, from the sensations experienced.

The regularity of the auricular systole was immediately restored by a new repletion of the heart.

This experiment having been repeated several times, we permited all the air to escape from the right ventricle and auricular appendage. Immediately all contraction ceased, even after touching with the point of the scalpel. The rythmic pulsations recommenced with all their regularity as soon as the right heart was refilled anew. This experiment was repeated several times with the same result.

These observations were prolonged for thirty minutes. We then directed our attention to the left heart. The auricular appendage distended with air did not contract spontaneously: it only did so under the influence of tickling with a soft point, and even then it only manifested fibrillary (fibrillaires) contractions, not extending over the entire surface.

The wall of the left ventricle, very hard, contracted upon itself, not at all distended by the insufflation, gave no sign of contractility.

Having, a little later, filled afresh the right heart (two hours and a half after the execution), we obtained no spontaneous contraction of the ventricle, nor of the auricular appendage. The latter always contracted but feebly, and not in its whole extent, when the surface was lightly scraped.

Several times, in the course of various experiments upon dogs dead of hæmorrhage, and recently upon a rabbit killed with this intention, by section of the neck, I have seen the contractions of the left heart, and also those of the right heart recommence spontaneously after their repletion with air, even after they had ceased for some minutes. But in consequence of conditions which I shall presently narrate, I have never seen these contractions preserve their rythmical character so long as in the criminal of whom I have just spoken. Always, after a few regular contractions, and especially from the resumption of the systole, they were tremulous and interrupted or only extended over a portion of the appendage and the ventricle.

It is difficult to know, in the presence of the preceding facts, and in the absence of all information about the contents of the heart, what could be the value of the observation of Emmanuel Rousseau, related by M. Richelot, from the recollections of Rousseau (Œuvres de Hunter), French translation, Paris, 1843, 8vo, t. III, p. 148, in note), according to which observation he has seen the heart of a woman pulsate *twenty-four hours* after execution. It was principally one of the auricular appendages which acted.

The other cavities of the heart expanded and contracted upon themselves alternately, but their movement was more obscure than that of the appendage. " These pulsations, " says the author, " persisted for more than three hours.

During these experiments, referred to above, it was very clearly seen, after the repletion of the appendages and of the auricles by the air, how their muscular wall is embraced between two serous membranes, likewise thin, but tenacious : how, in places, the muscular fasciculi separated leave free spaces in which the two serous membranes, the endocardium and the pericardium are applied and agglutinated to each other. In consequence of this disposition the contents of the auricular appendage are only separated from the pericardiac cavity by the thickness of the transparent sheet resulting from the fusion of the two cardiac serous membranes, which, moreover, as I have shown elsewhere, are, by reason of their texture, the organs to which the heart owes, principally, its resistance to rupture by distension.

(TO BE CONTINUED IN NEXT NUMBER.)

